# ‘Disease, Disaster and Despair’? The Presentation of Health in Low- and Middle-Income Countries on Australian Television

**DOI:** 10.1371/journal.pone.0014106

**Published:** 2010-11-24

**Authors:** Michelle Imison, Simon Chapman

**Affiliations:** Sydney School of Public Health, University of Sydney, Sydney, New South Wales, Australia; CIET, Canada

## Abstract

**Background:**

In high-income nations mainstream television news remains an important source of information about both general health issues and low- and middle-income countries (LMICs). However, research on news coverage of health in LMICs is scarce.

**Principal Findings:**

The present paper examines the general features of Australian television coverage of LMIC health issues, testing the hypotheses that this coverage conforms to the general patterns of foreign news reporting in high-income countries and, in particular, that LMIC health coverage will largely reflect Australian interests. We analysed relevant items from May 2005 – December 2009 from the largest health-related television dataset of its kind, classifying each story on the basis of the region(s) it covered, principal content relating to health in LMICs and the presence of an Australian reference point. LMICs that are culturally proximate and politically significant to Australia had higher levels of reportage than more distant and unengaged nations. Items concerning communicable diseases, injury and aspects of child health generally consonant with ‘disease, disaster and despair’ news frames predominated, with relatively little emphasis given to chronic diseases which are increasingly prevalent in many LMICs. Forty-two percent of LMIC stories had explicit Australian content, such as imported medical expertise or health risk to Australians in LMICs.

**Significance:**

Media consumers' perceptions of disease burdens in LMICs and of these nations' capacity to identify and manage their own health priorities may be distorted by the major news emphasis on exotic disease, disaster and despair stories. Such perceptions may inhibit the development of appropriate policy emphases in high-income countries. In this context, non-government organisations concerned with international development may find it more difficult to strike a balance between crises and enduring issues in their health programming and fundraising efforts.

## Introduction

It has long been noted that news media are influential in the formation of community and political health agenda [1], [2]. The broadcast and print media in both Britain and Australia tend to health coverage dominated by clinical settings, technological interventions and an individualised view of illness [3], [4]. These observations hold principally for local health stories, since domestic news dominates national programming generally, and for those from other high-income countries. British press coverage of medical research has been shown to completely ignore scientific advances from low- and middle-income countries (LMICs) [5]. A comprehensive review of determinants of international news found the strongest predictors of coverage to be the degree of nations' economic interaction and the availability of suitable material from news agencies [6].

Commentators have protested for some time that news coverage from LMICs in high-income nations is limited, often inaccurate and thus unfair [7], [8]. Reviews of international news coverage and agency material about Africa demonstrate a focus on a limited range of countries, with stories mainly about war, violence and political instability [9], [10]. As these news agencies are based in the high-income world, LMICs are further disadvantaged in their ability to influence global news flows [11]. This is significant as the news media, particularly television, remain the most important information source about LMICs in high-income nations [12]. However, the medium tends to present problematic depictions: one study of audience responses summed up coverage of LMICs on British television as being about ‘squalor and safari’, offering either over-simplified impressions of these places, or exaggeratedly negative caricatures [13]. Further, while recognising the importance of television in shaping viewers' impressions of LMICs, media producers often perceive such content as a ratings risk [14].

It would seem intuitive that these general patterns of coverage will be similar for health news. However, research on news coverage of LMIC health is scarce; major international medical journals, in which many health stories covered by the mass media originate, have historically under-represented LMIC concerns in their pages [15]. The only systematic examination of neglected tropical disease coverage in the international English-language electronic media located just 113 articles from across a four-and-a-half year period [16].

This study describes Australian television coverage of LMIC health between May 2005 and December 2009. It also tests hypotheses that:

- the coverage of LMIC health issues on Australian television is narrow in both scope and context, and provides an unbalanced picture of health issues in the regions and nations presented and
- a principal determinant of coverage for LMIC health items on Australian television will be the presence of Australians as victims or health workers in LMICs, or because of action by Australians to assist patients from LMICs to come to Australia for treatment.

## Methods

We used the Australian Health News Research Collaboration (AHNRC) database (http://www.health.usyd.edu.au/AHNRC/index.html) which, since May 2005, has archived all health-related, free-to-air Sydney television news, current affairs and ‘infotainment’ programme items [17]. At the end of December 2009, the then-52 month database contained 21 704 stories. News items are selected for archiving when they explicitly mention health care facilities or providers, any health outcome or risk factor, disease or injury, or political commentary about health. In order to limit the parameters of the database, stories about broader ‘social determinants of health’ (such as poverty, housing and employment) and deaths or injuries caused by natural disasters, war, civil unrest or criminal activity are not included unless they contain explicit mention of health (such as more than incidental coverage of the involvement of medical services) [17]. Preventable injury, such as road trauma, is included.

Each story was classified as being about up to two of 21 broad content categories and up to four of 218 specific sub-categories of content [17], one of which is ‘LMIC health’. The WHO defines LMICs to include nations with gross national income per capita less than US$10 066 in 2004 [18]. Within this sub-sample, we noted the regions to which news items referred (Table 1) and the frequency with which diseases and health conditions were covered (Table 2). To test our second hypothesis, all items were assigned to one of five categories that related to the presence or absence of a ‘local (Australian) angle’ (Table 3). Where several categories appeared in any given story, the predominant emphasis was chosen. To test the reliability of the principal coder's (MI) allocation, 40 items were randomly selected and three other coders categorised each against the definitions shown in Table 3.

**Table 1:** Regions and nations covered in 923 Australian television health news, current affairs and magazine reports about LMICs, May 2005 – December 2009.

Regions	Occurrences (%)
Global	88 (9.5)
Australia	286 (31.0)
Asia	506 (54.8)
Middle East and North Africa	118 (12.8)
Africa	113 (12.2)
Latin America and the Caribbean	71 (7.7)
Eastern Europe/former Soviet states	56 (6.1)
The Pacific	51 (5.5)

**NB: counts sum to more than 923, as some clips covered more than one region.**

**Table 2:** Diseases and conditions covered in 923 Australian television health news, current affairs and magazine reports about LMICs, May 2005 – December 2009.

Broad categories	Occurrences (%)
Communicable disease	359 (38.9)
Injury	204 (22.1)
Child health	191 (20.7)
Public health	138 (15.0)
General health	53 (5.7)
Chronic (non-communicable) disease and risk factors	30 (3.3)
Environmental health	22 (2.4)
Elective therapies/treatments	19 (2.1)
Health consequences of disasters	12 (1.3)
**Specific diseases/conditions/procedures**	
Surgery	170 (18.4)
H5N1 avian influenza (‘bird flu’)	159 (17.2)
Transport injury (bus, train and boat)	84 (9.1)
Unusual births	83 (9.0)
HIV/AIDS	81 (8.8)
Consumer product safety (food, toys and textiles)	70 (7.6)
Industrial and construction incidents	38 (4.1)
H1N1/A influenza (‘swine flu’)	32 (3.5)
Medical research	28 (3.0)
Pharmaceuticals	24 (2.6)
Malaria	22 (2.4)
Health care	20 (2.2)
Cholera	19 (2.1)
Polio	19 (2.1)
Unintentional injury	18 (2.0)
Fire	17 (1.8)
Birth control	14 (1.5)
Medical tourism	14 (1.5)
Industrial waste/chemical spills	13 (1.4)
Vaccination	13 (1.4)
Animal attacks	12 (1.3)

**NB: counts sum to more than 923, as some items cover more than one disease and/or condition.**

**Table 3:** Frequency of LMIC health news categories with Australian reference point in 923 Australian television health news and current affairs stories, May 2005 – December 2009.

News category	Occurrences (%)
Australians experiencing health problems in LMICs	105 (11.4)
Australians at risk because of health problems originating in LMICs	83 (9.0)
Australia/Australians assisting in LMICs	77 (8.3)
Individuals from LMICs brought to Australia for health care	123 (13.3)
LMIC-only stories (not involving any of above)	535 (58.0)

## Results

The kappa statistic for the reliability of coding in Table 3 was 0.76 (95% CI, 0.68 – 0.83), indicating a substantial level of agreement [19]. Items about LMICs (*n* = 923) constituted 4.3% of all stories, ranking LMIC health ninth among all news categories. In each table, counts sum to more than the total number of items as individual stories often feature more than one region or disease/health condition classification.

Table 1 shows regions covered during the study period. Middle Eastern and North African coverage was dominated by Egypt and Turkey largely due to, respectively, a bus crash involving Australian tourists and the avian influenza outbreak of 2005/06. As has been observed for UK press coverage [20], the Australian television presentation of health issues in Africa was heavily skewed toward larger, Anglophone former colonies. By contrast, 15 smaller countries received fewer than five mentions each. Coverage of Latin America and the Caribbean was also dominated by a few nations, with the majority of countries appearing fewer than five times. This large and diverse continent is almost absent from Australian television news [21]. Reporting on health in former Soviet bloc nations was dominated by stories about Russia heavily consonant with an image of the country's repressive past and crumbling present: chemical spills, industrial accidents and alcohol-related social harm. Similarly, ten of the eleven stories from Poland related to a multiple-fatality building collapse and the coverage of Georgia (two stories) was about a demolition-site accident and the brutal treatment of disabled children.

Consistent with a previous report on news neglect [22], the Pacific was the least-mentioned region, despite comprising Australia's closest neighbours. Papua New Guinea dominated coverage. All stories from the Solomon Islands and Nauru, and all but one from Fiji, related to tales of patients (mostly children) needing life-saving or -improving surgery unavailable in their home countries being brought to Australia to receive medical attention.

The relatively significant representation of Indonesia (*n* = 93) and Bangladesh (*n* = 67) both were largely due to specific stories: avian influenza and the separation of conjoined twins (in Australia), respectively. In contrast, the ten least-represented Asian countries had, collectively, less television exposure than any of the five most-covered Asian nations individually. These ten were either those – such as Laos or Cambodia – that are not yet economically or strategically central to Australia or (like Tibet) nations with which most Australians would have little or no familiarity.

Table 2 shows health issues from LMICs broadcast on Australian television, in broad health category aggregations as well as specific issues which merited more than ten stories. The pattern evident here is generally consonant with what has been termed a ‘disease, disaster and despair’ focus [23]. Communicable diseases were the most-reported broad category of health and illness; of such stories, infectious conditions that threatened Australia, and which originated in LMICs (like variant influenzas), were prominent. Coverage of HIV/AIDS, in particular, often featured vignettes and images of suffering and hopelessness. Tuberculosis and malaria, the two other diseases targeted by the Global Fund and mentioned in the Millennium Development Goals, together received only one-third of the television coverage given to HIV/AIDS.

Injury is by far the leading domestic health news category on Australian television [17]. Similarly, in reportage of LMICs, transport accidents, building collapses, maritime loss of life and animal attacks were prevalent, but without the perspective provided by the far more diverse range of health coverage offered in the Australian domestic context. This emphasis and absences underscore a sense that LMICs are unsafe places. As in high-income nations, stories from LMICs about children and child health were also favoured in Australian television coverage [4], [17]. The largest category of these related to children brought to Australia for surgery to either repair damage done by injury or rectify a birth defect (*n* = 115).

The broad category ‘public health’ comprised 15% of all stories and the majority of coverage here (54%) concerned the safety of Chinese-made goods, particularly toys treated with lead-based paints (2007) and melamine-tainted milk products (2008). All stories about toy safety, and over half of those about food safety, pertained either partly or wholly to Australia and the domestic health consequences of these goods' importation.

Australian television coverage of health in LMICs paid scant attention to non-communicable diseases. For instance, of only five obesity stories, one concerned an Australian living in Cambodia, another was a ‘freak story’ [24] dealing with the weight-loss efforts of a morbidly obese Mexican and two related to the pharmaceutical potential of a traditional Chinese remedy as a treatment for obesity. Only one story – a documentary about obesity throughout the world – dealt in any way with the experience of obesity in LMICs.

Table 3 shows the frequency of news categories, with or without reference to Australia. There were 388 stories (42%) relating explicitly to Australian involvement in, or action for, health in LMICs.

## Discussion

These findings broadly corroborate previous research on coverage of general foreign news: specifically, that it can be over-determined by cultural proximity to, and thus perceived interest for, audiences in high-income nations [25]; that health news from nations of economic and political significance is more likely to be broadcast on domestic television [26]; and that, although LMICs are now accorded increased media exposure, broadcasters tend to follow a relatively limited agenda of stories from such nations [13].

The patterns of news coverage of regions and countries follow Australia's perceived national interests: Asia, the world's most populous region and that in which Australia is located, was also the most-frequently covered and China, Australia's second-largest trading partner after Japan, was by far the most-mentioned nation. Australia, or Australian citizens and health workers, often figure in health news from LMICs, suggesting that this kind of domestic involvement is required for newsworthiness. In the case of the least-newsworthy nations, items tended to relate to the dramatic (multiple-fatality bus crashes in Egypt, Guatemala and Panama) or to feature extraordinary images (an explosion at a Puerto Rican petrol refinery that killed several people). Mexico received greater exposure, but over half of the relevant stories (*n = *22) concerned its status as the country of origin for the 2009 swine (H1N1) influenza outbreak, and the potential threat to Australia and other nations.

Two other trends stand out in the coverage described. First, the strong interest in certain countries while others were virtually or entirely ignored, and the narrow range of issues covered, is broadly consistent with previous findings about Australian news coverage of humanitarian crises and the media's focus on a small number of concerns at any one time [21]. Second, as in high-income nations, rates of non-communicable diseases and some of their principal risk factors are now also among the leading causes of morbidity and mortality in LMICs [27], [28]. Their omission from this dataset would appear a serious oversight. The patterns of LMIC health coverage outlined in this paper are likely to be explained by television professionals' editorial judgements that they are simply catering to the well-researched preferences of Australian audiences for news about issues of personal relevance and interest, ideally with arresting images [14], [29]. Chronic disease offers fewer of these opportunities than do stories of acute suffering.

We believe there are several broad implications arising from the patterns of coverage described: for individuals as both citizens assessing the appropriateness of government foreign policy toward LMICs and as potential private donors; and for domestic non-government organisations (NGOs) concerned with international development.

News media coverage shapes community perceptions. The emphases and neglects we have described are unlikely to assist momentum toward the ‘rational allocation’ of resources in development assistance for health [30] in LMICs, with public attention continually being drawn to a seemingly unchanging menu of newsworthy graphic incidents, disasters, pestilences, plagues and the ‘rescue’ of sick, usually young, individuals, often by Australian medical expertise. With the chronic illnesses now leading national disease burdens in many LMICs rarely covered, existing patterns of news may condition public expectations that government development assistance policy should broadly align with the ‘typical’ health problems in LMICs as consumed by Australian television viewers. Emergency relief, support for the control of infectious diseases and beneficence toward identified individuals would seem likely to endure as public priorities for government funding. By contrast, the low news profile of efforts to improve health-related infrastructure in LMICs, build and sustain public health capacity, reform public health law and other long-term ‘upstream’, population-focused initiatives is likely to provide little incentive for election-conscious governments to increase their support in such areas.

This phenomenon can reach its apotheosis when suffering children are featured: their appearance both reinforces news consumers' self-image as generous and compassionate, while also strengthening existing impressions of LMICs as poor and serially helpless [31]. A combination of vulnerability, the perceived unfairness of injury or serious illness to a young child and their photogenicity makes them ideal ‘talent’ in an image- and emotion-driven medium such as television [32]. These situations exemplify the ‘Rule of Rescue’: a moral imperative to prioritise the saving of specific individuals facing avoidable death in situations that horrify the onlooker and thus demand intervention [33].

About one-third of stories about child health in our dataset (64 of 191) were concerned with the surgical separation of conjoined twin girls discovered at an orphanage in Bangladesh and brought to Australia by a charity. Although heart-warming to see the extent to which the media and the nation expressed their care and financial concern for the children, the prominence of this story is inexplicable without reference to Australian self-interest. All news items mentioned the brilliance of the surgical team and the generosity of the Australian public. Notably absent across our sample was any coverage of the broader and much more televisually-mundane problems experienced by countless, but unknown, children in Bangladesh and similar countries – problems that cannot conceivably be solved by bringing each child to Australia or another high-income nation for expensive, tertiary care. In a domestic news context, however, this is a ‘solution’ that works for both television producers and audiences, and ensures these types of stories receive such prominence. This media treatment effaces any complexity in the health profiles, disease determinants and health systems of LMICs.

There are important ways in which Australian television coverage of health in LMICs is neither accurate nor representative. Despite their growing burden of non-communicable diseases, there were no stories about some of the top ten causes of death and disability – for example, ischaemic heart disease or depression – in such countries. By way of comparison, as of April 30, 2010, 541 of the AHNRC database's then-22,537 items were about heart disease (2.4%) and another 336 stories (1.5%) concerned depression. Most of these were Australian domestic health stories. Many less well-recognised communicable diseases, such as Marburg virus, Ebola and Noma disease, seem to have been covered for their value as ‘exotic’ conditions: outside the experience of the Australian viewer, but consonant with an understanding of Africa (in which each of these occurred) as inherently ‘polluted’ and biologically dangerous.

Mass-media reportage is the pre-eminent source of information about disasters in LMICs for audiences in high-income nations, and a valuable trigger for NGO fundraising. However, these same agencies express an uneasy ambivalence about the focus of this kind of coverage, pointing to what they perceive as sensationalism, an over-emphasis on ‘Western’ contributions to disaster relief and an interest in dramatic catastrophes rather than enduring issues [34]. Many NGOs concede that they would likely continue a pragmatic policy of supplying the kind of images media outlets desire: strongly emotive, and portraying those affected by disasters – children, if possible – as deserving but destitute. Although potentially demeaning to their subjects, such representations are recognised as an effective fundraising tool [32], [35].

The coverage we examined may encourage such ambivalence, and entails several balancing acts for NGOs. Agencies need to promote their health-related programmes to domestic constituencies to create public profile and stimulate donations. It is difficult for NGOs to ignore the historic legacy of ‘disease, disaster and despair’ stories which have served them well financially in the past. However, agencies would also express some desire to further educate interested donors about the complexities of international health and the processes of development; thus many make available online extensive documentation about their activities in the health field and the organisational worldviews that underpin them.

However the general expectations in Australia's NGO sector about development work in health – as horizontal, integrated and long-term – are more difficult to market than concrete, vertical programmes that aim to quickly ‘fix’ identifiable diseases and relieve immediate suffering [36], [37]. The former approach can make the transparency so valued in current models of governance more challenging to achieve as obvious, discrete targets are harder to identify and financial commitments seldom result in quick, tangible ‘deliverables’. A good example of this dilemma is non-communicable diseases. The very concept may be counter-intuitive to a large part of the Australian television audience, with the ‘disease and despair’ they are accustomed to thinking of in relation to LMICs much more likely to be of the communicable variety – in part, because of the media presentation of these countries. Among the most fundamental of all the values underpinning the NGO sector is a broad commitment to development as a process centred on the needs of beneficiary communities and involving them as partners in programme selection, design and implementation [38]. The prominence of an Australian focus in LMIC health stories on domestic television serves instead to reinforce the opposite proposition: that development is a donor-driven process and, without external intervention, communities in LMICs would not survive.
